# Children and adolescents’ positive youth development qualities and internet addiction during the COVID-19 pandemic: A longitudinal study in China

**DOI:** 10.3389/fpsyt.2022.1068737

**Published:** 2023-01-11

**Authors:** Zhuo Wang, Binxue Hong, Yanyan Zhang, Ya Su, Minghui Li, Li Zhao, Peng Jia

**Affiliations:** ^1^Department of Chronic and Non-communicable Disease Control and Prevention, Sichuan Center of Disease Control and Prevention, Chengdu, China; ^2^Department of Health Policy and Management, West China School of Public Health, Sichuan University, Chengdu, China; ^3^Department of Sociology and Anthropology, Swarthmore College, Swarthmore, PA, United States; ^4^Healthcare Evaluation and Organization Analysis (HEOA) Group, West China School of Public Health and West China Fourth Hospital, Sichuan University, Chengdu, China; ^5^International Institute of Spatial Lifecourse Health (ISLE), Wuhan University, Wuhan, China; ^6^School of Resource and Environmental Sciences, Wuhan University, Wuhan, China

**Keywords:** children, positive youth development, Internet addiction, cohort, China, COVID-19, adolescents

## Abstract

**Backgrounds:**

Recent studies have shown that the qualities of children and adolescents’ positive youth development (PYD) enable them to cope with developmental challenges in an adaptive manner and maintain healthy functioning. During the COVID-19 pandemic, there is still a lack of reporting on changes in children and adolescents’ PYD qualities and Internet addiction and their relationship. This study investigated the association between PYD qualities and Internet addiction among the children and adolescents who have experienced the COVID-19 lockdown.

**Methods:**

A school-based cohort survey was launched in December 2019 (Wave 1, before COVID-19 lockdown) and followed up in June 2020 (Wave 2, after COVID-19 lockdown). The Chinese PYD scale (80 items, scoring 80–480) and Young’s Internet addiction test (20 items, scoring 20–100) were used to evaluate the children and adolescents’ PYD qualities and the degree of their Internet addiction, respectively. Cross-sectional regressions, longitudinal regressions, and cross-lagged panel model were used to examine the association between PYD qualities and Internet addiction.

**Results:**

7,985 children and adolescents completed both waves of surveys. Compared with children and adolescents before lockdown (Wave 1), their total PYD quality dropped from 4.99 to 4.96 after COVID-19 lockdown (Wave 2), and the mean score for Internet addiction rose from 35.56 to 36.16. Cross-sectional analysis showed that after controlling for basic characteristics such as age and gender, the total PYD quality of children and adolescents in two waves was negatively correlated with the degree of Internet addiction during the same period, with β of −6.10 and −6.95, respectively. Longitudinal analysis showed that after controlling for basic characteristics, children and adolescents’ total PYD quality in Wave 1 was negatively correlated with the Wave 2 of Internet addiction and the change between the two waves of Internet addiction, with β of −3.35 and −0.26, respectively. Cross-lagged panel models showed a negative bilateral relationship between total PYD quality and Internet addiction.

**Conclusion:**

During the COVID-19 pandemic, the qualities of children and adolescents’ PYD declined, which makes children and adolescents more vulnerable to Internet addiction. Therefore, it is necessary to widely implement programs in China that can comprehensively improve the qualities of children and adolescents’ positive development to prevent Internet addiction, especially after the blockade due to public health emergencies.

## 1. Introduction

The frequency and duration of Internet use by Chinese children and adolescents have been increasing over the past few decades (1). Internet provides children and adolescents with a platform to learn, play games, watch movies, use social media, and chat. It is beneficial to use the network. For example, surfing the internet may alleviate feelings of loneliness and enhance social connection (2). But excessive and uncontrolled usage may develop into Internet addiction (3–5), which is characterized by excessive or poorly controlled obsession and desire for Internet use (6). This could lead to conflicts in real life, such as self-injurious behavior, academic failure, impulsivity and obsessive-compulsive symptoms, insomnia, loneliness, and interpersonal conflict (7, 8), and these conflicts may further aggravate their Internet addiction (9). Furthermore, the Asian Adolescent Risk Behavior Survey, which investigated six Asian countries and found the Internet addiction rate ranging from 6.2 to 16.4%, showed that Internet addictive behavior was common among adolescents in Asian countries (10).

The outbreak of COVID-19 may further accelerate Internet use and its related problems in children and adolescents (11–14). In January 2020, the Ministry of Education of China issued notices to postpone the opening of spring semester of all primary and secondary schools, and required students to stay at home and study through the Internet, which lasted for more than 3 months. Various online education platforms launched free live courses for students, and the scale of users increased rapidly. Remote learning, by nature, demands longer time spent online. The increased use of the Internet for educational purposes could also enhance opportunities for other online activities, such as video games or social media. Children and adolescents around the world are spending more time online to easy negative experiences of social alienation (2). However, it has been proved that over-reliance on the Internet as a primary means of dealing with anxiety could lead to Internet addiction (9). In a period of isolation without the structural support offered by the school, teachers, and peer students, children and adolescents may be particularly vulnerable to Internet addiction and other behavioral problems faced with uncertainties and anxiety brought by the pandemic. Insofar as the world still continues to struggle with the pandemic and online remote learning may be readopted due to emergency, it is important for scholars to investigate the factors that contribute to potential Internet addiction and the mechanism through which the factors can lead to Internet addictions (15).

Besides Internet use pattern, there are other influencing factors for Internet addiction of children and adolescents (16). While some studies focus on risk factors such as permissive parenting style (17), unhealthy dietary and lifestyle behaviors, as well as depression and other negative emotions (18, 19), protective factors like positive youth development (PYD) qualities have attracted tremendous attention from researchers, who support that the key to preventing problem behavior is to focus on the children’s strengths rather than weaknesses (20). Highlighted as a potentially important factor that may support better health and social outcomes (21, 22), PYD assets emphasize on building children’s developmental plasticity, internal developmental assets and external developmental assets, enabling children and adolescents to cope with developmental challenges in an adaptive manner and maintain healthy functioning (23). A cross-sectional study revealed that PYD qualities mediates the relation between Internet addiction and depression (7). A longitudinal study showed that students’ overall PYD qualities were negatively correlated with Internet addiction behavior, while prosocial attributes were positively correlated with Internet addiction (24).

According to some studies, PYD assets can protect children and adolescents from Internet addiction by the processes of “buffering” and “compensating” (25–27). “Buffering” means that risk factors in the environment would have less influence on the behavior of children and adolescents with positive assets (25). For example, when faced with negative life events, children tend to solve the problems rather than escape into the online world. Also, children with the assets may be more determined so they could resist peer temptation to engage in problem behavior such as excessive Internet use. “Compensation” is described as a process whereby children possessing positive assets are able to engage in risk with less harmful consequences (25). For example, if the children and adolescents have already been in the context of excessive Internet use, those with PYD assets are less prone to be addicted.

While prior studies provide important insights concerning the association between children and adolescents’ PYD qualities and Internet addiction, little is known regarding how this relationship is manifested during COVID-19 lockdown, a situation where Internet use has increased significantly due to remote learning (28). Using a longitudinal study design, our study examines how children and adolescents’ PYD qualities influence Internet addiction in a southwest city in China before and during the COVID-19 lockdown. Based on the literature, we propose the following hypotheses.

Hypothesis 1: PYD qualities would have negative cross-sectional relationships with Internet addiction in both Wave 1 and Wave 2.

Hypothesis 2: PYD qualities in Wave 1 would have negative longitudinal relationships with Internet addiction in Wave 2.

Hypothesis 3: PYD qualities would have negative relationships with change in Internet addiction over time.

Hypothesis 4: PYD qualities would have negative bilateral relations with Internet addiction.

## 2. Materials and methods

### 2.1. Participants

The Chengdu Survey of Positive Child Development (CPCD) was a school-based cohort with children and adolescents from five randomly selected primary and secondary schools (grade one to grade nine) from central and suburb Chengdu, Sichuan province, western China (29). All students from the five schools were invited to participate. Two waves of investigation were carried out. The first wave survey was performed before the outbreak of COVID-19 pandemic, from 23 December 2019 to 13 January 2020. The second wave survey was conducted after the resumption of primary and secondary schools in Chengdu, from 16 June 2020 to 8 July 2020. More details about the Survey of Positive Child Development have been reported (30, 31).

The study was reported in accordance with STROBE (Strengthening the Reporting of Observational studies in Epidemiology). This project received ethical approval from the Ethics Committee of Sichuan University (K2020025). The participating schools, children and adolescents, and their parents provided written informed consent before participating in the survey.

### 2.2. Measures of key variables

#### 2.2.1. Outcome variable

The probability of Internet addictive behaviors of the participants was measured by Young’s Internet addiction test. There are 20 questions and each has five options to indicate the frequency of occurrence, from 1 (rarely) to 5 (always). The total score was 100, and a higher score indicates a higher degree of Internet addiction. Young’s Internet addiction test is widely applied and has shown good reliability and validity in prior research (8, 32). In this study, the Cronbach’s α for the scale ranged from 0.95 to 0.96.

#### 2.2.2. Exposure variable

The participants’ PYD qualities were measured by the Chinese PYD Scale, the reliability, and validity of which have been tested among Chinese adolescents (Supplementary Table 1) (33, 34). The Chinese PYD Scale has 15 independent subscales, reflecting different psychosocial abilities, with a total of 80 questions (35). The 15 subscales can further reflect four comprehensive indicators (cognitive-behavioral competence, prosocial attributes, positive identity, and general PYD). Each question has six options to express their attitude, from 1 (strongly disagree) to 6 (strongly agree). In this study, the Cronbach’s α for the scale ranged from 0.86 to 0.94.

#### 2.2.3. Covariates

The sociodemographic characteristics of the participants and their family were collected from two data sources. Age, gender, and grade were reported by the participants personally. Pocket money received per week, their parents’ age, education level, occupation, and family living condition and monthly income were collected from their caregivers (using a caregiver questionnaire). These viables were used as adjusted covariates in the analysis.

### 2.3. Statistical analysis

Descriptive analysis of the participants’ social demographic variables (including personal, parental, and family characteristics) was conducted. The total number and percentage were used to describe the classified data, and the mean and standard deviation were used to describe the continuous data. The difference of each variable between groups was tested by χ2 tests for categorical variables or *t*-tests for continuous variables. Paired samples *t*-tests were performed to compare the change of PYD qualities and the Internet addiction score before and during the COVID-19 pandemic. Correlational analysis (R Package “xtable”) of the two waves was also conducted to show association between PYD qualities and Internet addiction scores. Multiple regression analysis (R Package “stats”) was conducted to explore the association between personal PYD qualities and Internet addiction in cross-sectional survey. Longitudinal multiple regression analysis was used to explore the predictive ability of wave 1 personal PYD qualities to wave 2 Internet addiction and its change. The cross-lagged panel model (R Package “lavaan”) was used to explore the bilateral relationships between PYD qualities and the Internet addiction. R version 4.1.0 (2021-05-18) was used for data analyses. Significant level was 0.05.

## 3. Results

### 3.1. Participants’ characteristics

Before and during the COVID-19, 7,985 children and adolescents (average age 10.60 years) completed the first and second wave surveys, among which 51.65% were boys, 68.28% were primary school students and 63.23% lived in city. The average age of their fathers was 39.41 years (SD = 6.41 years) and their mothers was 36.86 years (SD = 5.91 years). Most of the participants’ parents’ education level was middle school, which was 40.05% for fathers and 38.71% for mothers. The monthly gross household income was 8.55 thousand yuan (SD = 5.57 thousand yuan) (Table 1).

**TABLE 1:** Wave one characteristics of the two waves participants in Chengdu Survey of Positive Child Development (CPCD).

Characteristics	*N* (%) or Mean ± SD						
	All (*n* = 7,985)	Boys (*n* = 4,124)	Girls (*n* = 3,861)	*P*-value (sex)	Primary school (*n* = 5,452)	Middle school (*n* = 2,533)	*P*-value (grade)
Age (year)	10.60 ± 2.18	10.60 ± 2.19	10.60 ± 2.18	1.00	9.47 ± 1.59	13.00 ± 0.92	<0.01**
Sex							0.04*
Boy	4,124 (51.65)	-	-		2858 (52.42)	1266 (49.98)	
Girl	3,861 (48.35)	-	-		2594 (47.58)	1267 (50.02)	
Grade				0.04*			
Primary school	5,452 (68.28)	2,858 (69.30)	2,594 (67.18)		-	-	
Middle school	2,533 (31.72)	1,266 (30.70)	1,267 (32.82)		-	-	
Residence				0.19			<0.01**
Urban	5,049 (63.23)	2,627 (63.70)	2,420 (62.68)		3555 (65.21)	1492 (58.90)	
Rural	2,824 (35.37)	1,426 (34.58)	1,398 (36.21)		1816 (33.31)	1008 (39.79)	
Father’s age (year)	39.41 ± 6.19	39.30 ± 6.29	39.60 ± 6.08	0.03*	38.20 ± 5.90	42.10 ± 5.93	<0.01**
Mother’s age (year)	36.86 ± 5.91	36.80 ± 5.91	36.90 ± 5.92	0.25	35.60 ± 5.47	39.60 ± 5.88	<0.01**
Father’s highest educational level				0.03*			<0.01**
Primary school or below	579 (7.25)	301 (7.30)	278 (7.20)		303 (5.56)	276 (10.90)	
Middle school	3,198 (40.05)	1,585 (38.43)	1,613 (41.78)		2052 (37.64)	1146 (45.24)	
High school	1,948 (24.40)	1,007 (24.42)	941 (24.37)		1388 (25.46)	560 (22.11)	
Vocational or technical school	771 (9.66)	422 (10.23)	349 (9.04)		596 (10.93)	175 (6.91)	
University degree and above	1,248 (15.63)	670 (16.25)	578 (14.97)		935 (17.15)	313 (12.36)	
Mother’s highest educational level				0.05			<0.01**
Primary school or below	872 (10.92)	456 (11.06)	416 (10.77)		424 (7.78)	448 (17.69)	
Middle school	3,091 (38.71)	1,529 (37.08)	1,562 (40.46)		2000 (36.68)	1091 (43.07)	
High school	1,810 (22.67)	951 (23.06)	859 (22.25)		1332 (24.43)	478 (18.87)	
Vocational or technical school	814 (10.19)	423 (10.26)	391 (10.13)		626 (11.48)	188 (7.42)	
Bachelor or above	1,157 (14.49)	626 (15.18)	531 (13.75)		892 (16.36)	265 (10.46)	
Number of siblings	0.87 ± 1.01	0.88 ± 1.06	0.87 ± 0.96	0.60	0.89 ± 1.073	0.85 ± 0.86	0.02*
Family monthly income (K yuan)	8.55 ± 5.57	8.76 ± 5.67	8.33 ± 5.45	<0.01**	8.73 ± 5.65	8.17 ± 5.38	<0.01**

*P*-values tested the differences in each variable between sexes (boy and girl) and grades (primary and middle school), and were based on χ^2^ tests for categorical variables or *t*-tests for continuous variables.

*p < 0.05; **p < 0.01.

### 3.2. Reliability of measurements and correlational analyses

Supplementary Table 2 described PYD Scale and Young’s Internet addiction test in detail, and showed the good reliability of the measurements in the CPCD. The mean inter-item correlation and item-total correlation values were acceptable. In two Waves, the mean inter-item correlation of higher order factors of the PYD Scale ranged from 0.56 to 0.81, and the mean inter-item correlation of primary factors ranged from 0.47 to 0.83.

Supplementary Table 3 showed the correlations between all measurements in this study. The analyses showed PYD higher-order factors were positively correlated with each other, while PYD higher-order factors were negatively correlated with Internet addiction scores.

### 3.3. Changes of PYD qualities and Internet addiction before and during the COVID-19 pandemic

Changes of PYD qualities and Internet addiction before and during the COVID-19 pandemic were presented in Table 2. It was found that the value of cognitive-behavioral competence, prosocial attributes, positive identity, general PYD, and total PYD of the participants in the second wave decreased slightly compared with wave 1, in which positive identity, general PYD, and total PYD decreased significantly (*P* < 0.01), which ranged from 0.004 for prosocial attributes to 0.045 for general PYD. The average score of positive identity (4.80 in Wave 1 and 4.76 in Wave 2) was the lowest, while that of prosocial attributes (5.05 in Wave 1 and 5.04 in Wave 2) was the highest.

**TABLE 2:** PYD qualities and Internet addiction test scores among Chinese children and adolescents at two waves.

Items	Wave 1		Wave 2		Test effect			
	Mean 1	SD 1	Mean 2	SD 2	Mean 2–Mean 1	95% C.I.	*t*	*P*
**PYD attributes**								
Cognitive-behavioral competence	5.02	0.75	5.00	0.81	−0.02	−0.04∼0.0009	−1.87	0.06
Prosocial attributes	5.05	0.82	5.04	0.86	−0.004	−0.025∼0.017	−0.39	0.699
Positive identity	4.80	0.93	4.76	0.97	−0.038	−0.06∼−0.016	−3.41	0.00065**
General PYD	5.01	0.71	4.97	0.77	−0.045	−0.06∼−0.028	−5.26	<0.01**
Total PYD	4.99	0.69	4.96	0.75	−0.033	−0.049∼−0.017	−4.01	<0.01**
**Internet addiction**								
Young’s Internet addiction test	35.56	14.74	36.16	15.23	0.60	0.28∼0.93	3.68	0.0002**

PYD, Positive youth development; C.I., Confidence interval.

*p < 0.05; **p < 0.01.

The Internet addiction score of participants in the second wave (mean = 36.16, SD = 15.23) was statistically 0.60 higher than that in the first wave (mean = 35.56, SD = 14.74) (*P* < 0.001).

### 3.4. Association between children and adolescents’ PYD qualities and Internet addiction

Table 3 showed that Wave 1 PYD qualities significantly predicted Internet addiction in Wave 1, including cognitive-behavioral competence (β = −4.71, *P* < 0.01), prosocial attributes (β = −3.73, *P* < 0.01), positive identity (β = −3.67, *P* < 0.01), general PYD (β = −6.12, *P* < 0.01), and total PYD (β = −6.10, *p* < 0.01), adjusted for age, sex, grade, and residence. Similar findings were observed for the Wave 2 PYD qualities and Internet addiction test, including cognitive-behavioral competence (β = −5.47, *P* < 0.01), prosocial attributes (β = −4.60, *P* < 0.01), positive identity (β = −4.90, *P* < 0.01), general PYD (β = −6.72, *P* < 0.01), and total PYD (β = −6.95, *P* < 0.01).

**TABLE 3:** Cross-sectional multiple regression analyses for Internet addiction.

Model	Predictors	Young’s Internet addiction test Wave 1					Young’s Internet addiction test Wave 2				
		β	SE	*t*	*R* ^2^	*F*	β	SE	*t*	*R* ^2^	*F*
1	Age (year)	1.00	0.13	7.91**	0.145	112.4	1.24	0.13	9.59**	0.16	125.8
	Sex^a^	-3.71	0.33	-11.09**			-3.48	0.34	-10.12**		
	Grade^b^	6.87	0.57	12.13**			6.90	0.58	11.87**		
	Residence^c^	0.81	0.36	2.25*			1.66	0.37	4.50**		
	Father’s age (year)	-0.02	0.05	-0.37			0.10	0.05	1.94		
	Mother’s age (year)	0.05	0.05	1.02			-0.10	0.05	-1.83		
	Father’s highest educational level^d^	-0.22	0.19	-1.18			-0.27	0.19	-1.40		
	Mother’s highest educational level^d^	-0.33	0.19	-1.78			-0.35	0.19	-1.81		
	Number of siblings^e^	-0.04	0.37	-0.11			0.44	0.38	1.15		
	Family monthly income^f^	0.61	0.35	1.74			0.77	0.36	2.14*		
2	**PYD attributes**										
	Cognitive-behavioral competence	-4.71	0.22	-21.43**	0.20	328.7	-5.47	0.21	-26.50**	0.24	344.5
	Prosocial attributes	-3.73	0.20	-18.34**	0.18	300.0	-4.60	0.20	-23.51**	0.22	314.9
	Positive identity	-3.67	0.18	-20.46**	0.19	319.1	-4.90	0.17	-28.20**	0.25	362.9
	General PYD	-6.12	0.23	-26.60**	0.23	386.6	-6.72	0.21	-31.41**	0.27	400.8
	Total PYD	-6.10	0.24	-25.87**	0.22	377.7	-6.95	0.22	-31.68**	0.27	404.1

PYD, Positive youth development.

In model 1, control variables were characteristics in Wave 1.

In model 2, age, sex, grade and residence were statistically controlled in Young’s Internet addiction test Wave 1 model; age, sex, grade, residence and income were statistically controlled in Young’s Internet addiction test Wave 2 model; measures of PYD in Wave 1 and Wave 2 were included as predictors to predict Internet addiction in Wave 1 and Wave 2, respectively.

*p < 0.05; **p < 0.01.

^a^1 = Male, 2 = Female; ^b^1 = Primary school, 2 = Middle school; ^c^1 = Urban, 2 = Rural; ^d^1 = Primary school or lower, 2 = Junior secondary school, 3 = Senior secondary school, 4 = Diploma, 5 = Undergraduate or higher; ^e^1 = Only-child, 2 = Non-only-child; ^f^1 = Gross household income (Monthly) was less than 8 K yuan, 2 = Gross household income (Monthly) was more than or equal to 8 K yuan.

Supplementary Table 4 showed the sensitivity analysis of the cross-sectional multiple regression. Since children and adolescents might have different psychopathology, we did a sensitivity analysis. We categorized the covariate “age” into children (age less than 10 years) and adolescents (10 years or above), and the result showed that the association direction of the key variables remained the same and the coefficient is still significant (see Supplementary Table 4).

Longitudinal multiple regression analyses were conducted to test the influence of Wave 1 PYD qualities on Wave 2 Internet addiction and its changes (see Table 4). After controlling for the Wave 1 covariates (age, sex, grade, residence, father’s age, mother’s age, and Wave 1 Internet addiction), it was found that PYD qualities in Wave 1 significantly predicted Wave 2 Internet addiction, including cognitive-behavioral competence (β = −2.32, *P* < 0.01), prosocial attributes (β = −2.15, *P* < 0.01), positive identity (β = −2.25, *P* < 0.01), general PYD (β = −3.26, *P* < 0.01), and total PYD (β = −3.35, *P* < 0.01).

**TABLE 4:** Longitudinal multiple regression analyses for Internet addiction.

Model	Predictors	Young’s Internet addiction test Wave 2					Young’s Internet addiction test change between Wave 1 and Wave 2 (Mean 2–Mean 1)				
		β	SE	*t*	R^2^	*F*	β	SE	*t*	*R*^2^ Change	*F* Change
1	Age (year)	0.72	0.11	6.41**	0.38	371.9	0.25	0.13	1.94	0.23	175.1
	Sex^a^	-1.52	0.30	-5.10**			0.23	0.33	0.69		
	Grade^b^	3.27	0.50	6.49**			0.04	0.57	0.06		
	Residence^c^	1.24	0.32	3.90**			0.85	0.36	2.38*		
	Father’s age (year)	0.11	0.04	2.49*			0.11	0.05	2.37*		
	Mother’s age (year)	-0.12	0.05	-2.75**			-0.15	0.05	-2.91**		
	Father’s highest educational level^d^	-0.15	0.16	-0.93			-0.05	0.19	-0.26		
	Mother’s highest educational level^d^	-0.17	0.16	-1.05			-0.02	0.19	-0.09		
	Number of siblings^e^	0.46	0.33	1.41			0.48	0.37	1.29		
	Family monthly income^f^	0.45	0.31	1.45			0.16	0.35	0.46		
	Young’s Internet addiction test Wave 1	0.53	0.01	48.79**							
2	**PYD attributes**										
	Cognitive-behavioral competence	-2.32	0.20	-11.31**	0.39	535.7	-0.01	0.22	-0.07	0.002	3.99
	Prosocial attributes	-2.15	0.19	-11.54**	0.39	536.8	-0.31	0.21	-1.53	0.003	4.57
	Positive identity	-2.25	0.17	-13.56**	0.40	547.0	-0.46	0.18	-2.54*	0.003	5.60
	General PYD	-3.26	0.22	-14.75**	0.40	553.8	-0.15	0.24	-0.64	0.002	4.089
	Total PYD	-3.35	0.22	-14.88**	0.40	554.6	-0.26	0.24	-1.06	0.003	4.27

PYD, Positive youth development.

In model 1, control variables were characteristics in Wave 1.

In model 2, age, sex, grade, residence, father’s age, mother’s age and Young’s Internet addiction test in Wave 1 were statistically controlled in Young’s Internet addiction test Wave 2 model; residence, father’s age and mother’s age were statistically controlled in Young’s Internet addiction test change model; measures of PYD in Wave 1 were included as predictors in the model separately.

*p < 0.05; **p < 0.01.

^a^1 = Male, 2 = Female; ^b^1 = Primary school, 2 = Middle school; ^c^1 = Urban, 2 = Rural; ^d^1 = Primary school or lower, 2 = Junior secondary school, 3 = Senior secondary school, 4 = Diploma, 5 = Undergraduate or higher; ^e^1 = Only-child, 2 = Non-only-child; ^f^1 = Gross household income (Monthly) was less than 8 K yuan, 2 = Gross household income (Monthly) was more than or equal to 8 K yuan.

The results of the effect of Wave 1 PYD qualities on the changes of Internet addiction between the two waves showed that cognitive-behavioral competence (β = −0.01, *P* > 0.05), prosocial attributes (β = −0.31, *P* > 0.05), positive identity (β = −0.46, *P* < 0.05), general PYD (β = −0.15, *P* > 0.05), and total PYD (β = −0.26, *P* > 0.05) were negatively correlated with the changes of Internet addiction score, adjusted for residence, father’s age, and mother’s age (see Table 4).

Supplementary Table 5 showed the sensitivity analysis of the longitudinal multiple regression. And the result showed that the association direction of the key variables remained the same and the coefficient was still significant (see Supplementary Table 5).

**FIGURE 1:**
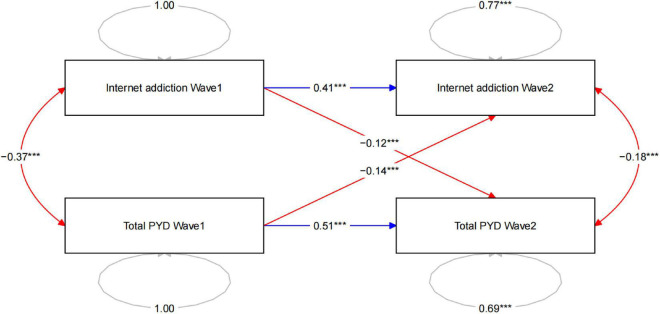
Cross-lagged panel model between positive youth development (PYD) qualities and the Internet addiction. Internet addiction Wave 1: Internet addiction scores before the COVID-19 lockdown; Internet addiction Wave 2: Internet addiction scores during the COVID-19 lockdown; Total PYD Wave 1: Total PYD score before the COVID-19 lockdown; Total PYD Wave 2: Total PYD score during the COVID-19 lockdown. The two-way arrow indicates the result of correlation analysis; the one-way arrow indicates the result of path analysis; the correlation coefficient and regression coefficient are a standardized estimations. ***p < 0.001.

The cross-lagged panel model showed that higher total PYD score at wave 1 was significantly associated with lower Internet addiction score at wave 2 (β = −0.14, *P* < 0.001). Higher Internet addiction score at wave 1 was also significantly associated with lower total PYD score at wave 2 (β = −0.12, *P* < 0.001). Total PYD score at wave 1 were positively related to the total PYD score at wave 2 (β = 0.51, *P* < 0.001), and previous Internet addiction score was also positively related to Internet addiction score during the COVID-19 lockdown (β = 0.41, *P* < 0.001) (Figure 1).

## 4. Discussion

This study, as a longitudinal study on primary and secondary school students in Chengdu before and during the pandemic, has made an important theoretical contribution to our understanding of the association between children and adolescents’ PYD qualities and Internet addiction. It is a two-round investigation under the circumstances of school blockade, 3 months of online teaching and social isolation, to explore the interaction between Chinese children and adolescents’ PYD qualities and Internet addiction before and during the COVID-19 pandemic. It was found that, compared to before the outbreak, the PYD qualities of primary and secondary school students decreased slightly and the degree of Internet addiction increased after the outbreak. Cross-sectional data analysis, longitudinal data analysis, and cross-lagged panel model all showed that the PYD qualities of students were negatively correlated with Internet addiction, that was, children and adolescents with higher PYD qualities had a lower degree of Internet addiction.

Theoretically speaking, children, and adolescents’ PYD qualities is relatively stable in “normal” or “COVID-free” periods, which comprehensively reflected their long-term personal experience, environmental conditions, and cultural background (33). However, in special circumstances, such as in the challenging era of COVID-19, children and adolescents’ adjustment may be affected. Several studies have reported that nationwide closures of schools and colleges have negatively impacted over 91% of the world’s student population, and that depression, loneliness, and anxiety were on the rise among young people (28, 36–39). The comparative analysis before and after the pandemic in our study showed some of the children’s PYD qualities (positive identity and general PYD) decreased. This suggested that the pandemic may affect children and adolescents’ positive and healthy cognition of themselves, optimism about the future, positive relationship with significant others, the ability to overcome adversity and challenges, and their ability to understand and manage emotions. The immediate cause may be due to disruptions of children and adolescents’ living and learning patterns during the outbreak of the pandemic. Moore et al. reported that the pandemic resulted in lower level of physical activity, less outdoor activities, more sedentary behaviors (including leisure screen time), and more sleep time (40). All of these factors may affect the PYD qualities of children and adolescents during COVID-19 pandemic.

Children and adolescents with good qualities of positive development may significantly reduce the risk for Internet addiction. The cross-sectional data analysis of this study showed that after controlling for basic characteristics such as age, gender, grade, residence, parental age, and parental education, the total PYD in the two waves was negatively correlated with the degree of Internet addiction during the same period. Longitudinal data analysis showed that, after controlling for basic characteristics, the children and adolescents’ total PYD in wave 1 was negatively correlated with the degree of Internet addiction in wave 2. Cross-lagged panel models also showed a negative bilateral relationship between total PYD quality and Internet addiction. This study highlighted that children and adolescents’ PYD qualities can affect the degree of Internet addiction. First, these findings were consistent with limited literature reports that higher PYD qualities can reduce Internet addiction and other adverse events, such as emotional capacity, spirituality, and resilience, showing strong, and stable protective effects against Internet addiction (41, 42). Secondly, the four PYD higher-order factors of children and adolescents, namely, cognitive-behavioral competence, prosocial attributes, positive identity, and general PYD, were important protective factors for Internet addiction before and during the pandemic. Cross-sectional and longitudinal data analysis showed that the order of influence on children and adolescents’ Internet addiction was general PYD, cognitive-behavioral competence, positive identity, and prosocial attributes. The longitudinal data analysis also showed that positive identity had the greatest impact on the changes in children and adolescents’ two Internet addiction degrees. This also provides practical significance and support for the widespread promotion of Chinese youth PYD projects. A good example to draw from is Hong Kong’s youth development framework project, Project P.A.T.H.S., which provided school educators with important knowledge about the important role of PYD qualities and life satisfaction in adolescent academic wellbeing, and aimed to enhance children and adolescents’ psychosocial competencies, and facilitating their holistic development (34, 43, 44). Third, after controlling for basic characteristics and the baseline degree of Internet addiction in wave 1, children and adolescents’ first wave of PYD qualities were negatively correlated with the second wave of Internet addiction, but there was no statistical correlation with the change of Internet addiction between the two waves. This may be related to the short interval between the two waves of investigation in this study (45), which deserves further exploration. Fourth, in addition to children and adolescents’ PYD qualities, other individual factors and family factors also played an important role in influencing children and adolescents’ Internet addiction. In the cross-sectional analyze, older, male, and urban-dwelling children and adolescents were likely to get higher Internet addiction scores, which was consistent with previous reports (46, 47). This may be partly due to the fact that adolescents with these characteristics have more access to the Internet and can engage in more online activities. In the longitudinal data analysis, participants with these characteristics, especially those with older father and younger mothers, might get higher scores as well, which indicated that parents played a role in the development of children and adolescents’ Internet addiction. Although the influence of parents was small in this study, it may be related to parent-child relationship, company of parents, and the way they educate their children, which needs further investigation (39, 48–50).

This study has several limitations. First, this is a short-term longitudinal study which cannot fully demonstrate the impact of COVID-19 pandemic on PYD qualities and Internet addiction among Chinese children and adolescents. Second, although the five schools in this study are randomly selected, the sample is concentrated in one city in China, so replication of this study in other regions is needed to verify the findings. Third, although this study showed that higher PYD qualities may reduce Internet addiction in children and adolescents, further study on the underlying mechanisms is needed. Despite these limitations, this study acts as a pioneer in that it provide timely empirical evidence to show the protective role of PYD qualities in children and adolescents’ Internet addiction, and further demonstrates the important impact of good PYD qualities on children in a period with high uncertainty and instability (51). Therefore, future studies can adopt a long-term and large-scale survey of children’s positive development to construct a more stable and comprehensive perspective to look at the association between PYD qualities and children and adolescents’ Internet addiction. We also suggest that future research should pay attention to the ramifications of lockdown on children and adolescents’ psychological wellbeing and design related interventions to help children and adolescents cope better with the stressful and uncertain transitions and prevent them from behavioral problems such as Internet addictions.

## 5. Conclusion

Overall, during the COVID-19 pandemic, the qualities of children and adolescents’ PYD had declined and the degree of Internet addiction increased. High PYD qualities can significantly reduce the degree of Internet addiction. Therefore, it is imperative to develop and implement targeted support to improve youth’s PYD qualities. As the ongoing pandemic affects many regions and countries around the world, findings of this research have implications beyond the case of China.

## Data availability statement

The data that support the findings of this study are available from the corresponding author upon reasonable request.

## Ethics statement

The studies involving human participants were reviewed and approved by Ethics Committee of Sichuan University (K2020025). Written informed consent to participate in this study was provided by the participants’ legal guardian/next of kin.

## Author contributions

LZ led data collection. ZW and BH performed data analysis. ZW prepared the initial draft of the manuscript. All authors made critical revisions and approved the final draft of the manuscript.
