# Supplementary material for: Children and adolescents’ positive youth development qualities and internet addiction during the COVID-19 pandemic: A longitudinal study in China
Source: Front Psychiatry. 2023 Jan 11;13:1068737. doi: 10.3389/fpsyt.2022.1068737 (PMC9875032; doi:10.3389/fpsyt.2022.1068737)
Supplement: Supplementary file 1 [file Table_1.docx]

Table S1. Description of Chinese Positive Youth Development Scale and Young's Internet addiction test

| Measurements | Higher-order  factors | Basic dimensions | No. of  variables | Brief Description |
| --- | --- | --- | --- | --- |
| **Chinese PYD Scale** | | | **80** | **6-point scale (1- strongly disagree to 6-strongly agree)** |
|  | Cognitive-behavioral competence | Cognitive competence | 6 | The ability of teenagers to think and solve problems |
|  |  | Behavioral competence | 5 | Whether adolescents can use verbal and nonverbal skills to promote self-confidence behavior |
|  |  | Self-determination | 5 | Whether teenagers can make age appropriate decisions |
|  | Prosocial attributes | Prosocial involvement | 5 | Whether teenagers have the opportunity to participate in Prosocial activities |
|  |  | Prosocial norms | 5 | Whether the teenagers understand and meet the expectations of society |
|  | Positive identity | Clear and positive identity | 7 | Whether teenagers have positive and healthy cognition of themselves |
|  |  | Beliefs in the future | 3 | Whether teenagers are optimistic about the future |
|  | General Positive youth development | Bonding | 6 | Positive relationships between adolescents and important others, healthy adults and positive peers |
|  |  | Resilience | 6 | The ability of young people to overcome adversity and challenges |
|  |  | Social competence | 7 | The ability of teenagers to interact with others in a healthy way |
|  |  | Recognition for positive behavior | 4 | Whether the good behavior of teenagers is appreciated by others, especially teachers |
|  |  | Emotional competence | 6 | Whether teenagers can understand and manage emotions |
|  |  | Moral competence | 6 | Whether teenagers are able to make reasonable moral decisions and act in a moral way |
|  |  | Self-efficacy | 2 | Whether teenagers believe in their ability and perceived ability can be used to achieve the goals set |
|  |  | Spirituality | 7 | Whether teenagers have the meaning of life and other attributes of existence |
| **Young's Internet addiction test** | | | **20** | - **5-point scale (1- rarely to 5- always)** |
|  |  | Internet addiction | 20 | The total score was 100. Whether they had the listed Internet addictive behaviors, including “After offline, eager to go online next time”, “feeling restless or irritable when attempting to cut down or stop on-line use”, etc. |

Note. PYD = Positive youth development.

Table S2. Reliability of Chinese Positive Youth Development Scale at two waves

| Items |  | Wave 1 | |  |  | Wave 2 | |
| --- | --- | --- | --- | --- | --- | --- | --- |
|  | α | Mean Inter-Item  Correlation | Mean Item-Total  Correlation |  | α | Mean Inter-Item  Correlation | Mean Item-Total  Correlation |
| **PYD primary factors** | | | | | | | |
| Cognitive competence | 0.93 | 0.47 | 0.81 |  | 0.93 | 0.50 | 0.83 |
| Behavioral competence | 0.93 | 0.47 | 0.76 |  | 0.93 | 0.50 | 0.77 |
| Self-determination | 0.93 | 0.48 | 0.75 |  | 0.93 | 0.51 | 0.77 |
| Prosocial involvement | 0.93 | 0.48 | 0.73 |  | 0.94 | 0.51 | 0.76 |
| Prosocial norms | 0.93 | 0.49 | 0.63 |  | 0.94 | 0.52 | 0.66 |
| Clear and positive identity | 0.93 | 0.48 | 0.76 |  | 0.93 | 0.51 | 0.78 |
| Beliefs in the future | 0.93 | 0.48 | 0.73 |  | 0.94 | 0.51 | 0.75 |
| Bonding | 0.93 | 0.48 | 0.68 |  | 0.94 | 0.51 | 0.71 |
| Resilience | 0.93 | 0.48 | 0.74 |  | 0.93 | 0.50 | 0.79 |
| Social competence | 0.93 | 0.48 | 0.76 |  | 0.94 | 0.51 | 0.76 |
| Recognition for positive behavior | 0.93 | 0.48 | 0.71 |  | 0.94 | 0.51 | 0.74 |
| Emotional competence | 0.93 | 0.48 | 0.76 |  | 0.94 | 0.51 | 0.76 |
| Moral competence | 0.93 | 0.48 | 0.75 |  | 0.93 | 0.51 | 0.76 |
| Self-efficacy | 0.93 | 0.49 | 0.67 |  | 0.94 | 0.52 | 0.67 |
| Spirituality | 0.93 | 0.50 | 0.52 |  | 0.94 | 0.54 | 0.54 |
| **PYD higher-order factors** | | | | | | | |
| Cognitive-behavioral competence | 0.88 | 0.60 | 0.48 |  | 0.90 | 0.64 | 0.52 |
| Prosocial attributes | 0.90 | 0.64 | 0.42 |  | 0.91 | 0.67 | 0.47 |
| Positive identity | 0.89 | 0.62 | 0.47 |  | 0.90 | 0.65 | 0.54 |
| General PYD | 0.88 | 0.59 | 0.54 |  | 0.89 | 0.62 | 0.57 |
| Total PYD | 0.86 | 0.56 | 0.55 |  | 0.88 | 0.60 | 0.58 |
| **Young's Internet addiction test** | 0.95 | 0.79 | 0.98 |  | 0.96 | 0.81 | 0.98 |

Note. PYD = Positive youth development.

Table S3. Correlational analyses at two waves

| Index | Measures | Correlations | | | | | | | | | | | | | | | | | | | | | |
| --- | --- | --- | --- | --- | --- | --- | --- | --- | --- | --- | --- | --- | --- | --- | --- | --- | --- | --- | --- | --- | --- | --- | --- |
|  |  | 1 | 2 | 3 | 4 | 5 | 6 | 7 | 8 | 9 | 10 | 11 | 12 | | 13 | 14 | 15 | 16 | 17 | 18 | 19 | 20 | 21 |
| 1 | Age(year) | — |  |  |  |  |  |  |  |  |  |  |  |  | |  |  |  |  |  |  |  |  |
| 2 | Sex ^a^ | 0.02 | — |  |  |  |  |  |  |  |  |  |  |  | |  |  |  |  |  |  |  |  |
| 3 | Grade ^b^ | 0.77** | 0.03** | — |  |  |  |  |  |  |  |  |  |  | |  |  |  |  |  |  |  |  |
| 4 | Residence ^c^ | 0.03* | 0.03* | 0.05** | — |  |  |  |  |  |  |  |  |  | |  |  |  |  |  |  |  |  |
| 5 | Father’s age (year) | 0.36** | 0.03* | 0.31** | 0.02 | — |  |  |  |  |  |  |  |  | |  |  |  |  |  |  |  |  |
| 6 | Mother’s age (year) | 0.39** | 0.02 | 0.33** | 0.02 | 0.83** | — |  |  |  |  |  |  |  | |  |  |  |  |  |  |  |  |
| 7 | Father’s highest educational level ^d^ | -0.20** | -0.03** | -0.15** | -0.01 | -0.18** | -0.17** | — |  |  |  |  |  |  | |  |  |  |  |  |  |  |  |
| 8 | Mother’s highest educational level ^d^ | -0.24** | -0.02 | -0.19** | -0.02 | -0.26** | -0.25** | 0.65** | — |  |  |  |  |  | |  |  |  |  |  |  |  |  |
| 9 | Number of siblings ^e^ | 0.02* | 0.04** | 0.02 | 0 | 0.15** | 0.16** | -0.16** | -0.19** | — |  |  |  |  | |  |  |  |  |  |  |  |  |
| 10 | Family monthly income ^f^ | -0.05** | -0.02* | -0.05** | 0.01 | -0.12** | -0.11** | 0.22** | 0.22** | -0.09** | — |  |  |  | |  |  |  |  |  |  |  |  |
| 11 | Wave 1 Cognitive-behavioral competence | -0.14** | 0.07** | -0.17** | -0.01 | -0.09** | -0.09** | 0.11** | 0.12** | -0.05** | 0.06** | — |  |  | |  |  |  |  |  |  |  |  |
| 12 | Wave 1 Prosocial attributes | -0.09** | 0.10** | -0.13** | 0 | -0.05** | -0.06** | 0.10** | 0.11** | -0.07** | 0.05** | 0.67** | — |  | |  |  |  |  |  |  |  |  |
| 13 | Wave 1 Positive identity | -0.19** | 0.02 | -0.21** | -0.01 | -0.10** | -0.11** | 0.14** | 0.15** | -0.07** | 0.05** | 0.72** | 0.64** | — | |  |  |  |  |  |  |  |  |
| 14 | Wave 1 General PYD | -0.17** | 0.06** | -0.19** | -0.01 | -0.09** | -0.10** | 0.12** | 0.13** | -0.07** | 0.06** | 0.84** | 0.70** | 0.75** | | — |  |  |  |  |  |  |  |
| 15 | Wave 1 Total PYD | -0.17** | 0.07** | -0.20** | -0.01 | -0.09** | -0.10** | 0.13** | 0.14** | -0.07** | 0.06** | 0.91** | 0.80** | 0.84** | | 0.97** | — |  |  |  |  |  |  |
| 16 | Wave 2 Cognitive-behavioral competence | -0.16** | 0.02* | -0.20** | -0.02 | -0.09** | -0.10** | 0.08** | 0.11** | -0.01 | 0.03* | 0.45** | 0.36** | 0.42** | | 0.47** | 0.49** | — |  |  |  |  |  |
| 17 | Wave 2 Prosocial attributes | -0.14** | 0.08** | -0.17** | -0.02 | -0.07** | -0.07** | 0.06** | 0.08** | -0.01 | 0.03* | 0.36** | 0.42** | 0.35** | | 0.42** | 0.44** | 0.70** | — |  |  |  |  |
| 18 | Wave 2 Positive identity | -0.23** | -0.01 | -0.24** | -0.01 | -0.12** | -0.12** | 0.10** | 0.13** | -0.03* | 0.04** | 0.42*f* | 0.34** | 0.52** | | 0.48** | 0.50** | 0.75** | 0.69** | — |  |  |  |
| 19 | Wave 2 General PYD | -0.19** | 0.02 | -0.20** | -0.01 | -0.09** | -0.10** | 0.08** | 0.11** | -0.01 | 0.03* | 0.44** | 0.38** | 0.45** | | 0.54** | 0.53** | 0.85** | 0.73** | 0.78** | — |  |  |
| 20 | Wave 2 Total PYD | -0.20** | 0.03* | -0.22** | -0.02 | -0.10** | -0.11** | 0.09** | 0.12** | -0.01 | 0.03** | 0.46** | 0.41** | 0.48** | | 0.54** | 0.54** | 0.91** | 0.82** | 0.86** | 0.97** | — |  |
| 21 | Wave 1 Young's Internet addiction test | 0.33** | -0.12** | 0.34** | 0 | 0.14** | 0.15** | -0.10** | -0.12** | 0.01 | -0.01 | -0.30** | -0.26** | -0.30** | | -0.36** | -0.36** | -0.27** | -0.26** | -0.29** | -0.29** | -0.31** | — |
| 22 | Wave 2 Young's Internet addiction test | 0.35** | -0.10** | 0.35** | 0.01 | 0.15** | 0.14** | -0.11** | -0.13** | 0.03* | -0.01 | -0.29** | -0.27** | -0.32** | | -0.36** | -0.36** | -0.35** | -0.32** | -0.39** | -0.40** | -0.41** | 0.59** |

Note. PYD = Positive youth development. * p < 0.05; ** p < 0.01.

^a^ 1 = male, 2 = female; ^b^ 1 = Primary school, 2 = Middle school; ^c^ 1 = Urban, 2 = Rural; ^d^ 1=Primary school or lower, 2=Junior secondary school, 3=Senior Secondary school3, 4=Diploma4, 5=Undergraduate or higher; ^e^ 1 = Only-child, 2 = Non-only-child; ^f^ 1 = Gross Household Income (Monthly) was less than 8 K yuan, 2 = Gross Household Income (Monthly) was more than or equal to 8 K yuan.

Table S4. Cross-sectional multiple regression analyses for Internet addiction (age as a binary variable)

| Model | Predictors | Young's Internet addiction test Wave 1 | | | | |  | Young's Internet addiction test Wave 2 | | | | |
| --- | --- | --- | --- | --- | --- | --- | --- | --- | --- | --- | --- | --- |
|  |  | β | SE | t | R2 | F |  | β | SE | t | R2 | F |
| 1 | Age (year)^a^ | 5.25 | 0.39 | 13.38** | 0.07 | 57.71 |  | 7.16 | 0.40 | 17.81** | 0.09 | 76.15 |
|  | Sex ^b^ | -3.59 | 0.35 | -10.31** |  |  |  | -3.37 | 0.36 | -9.46** |  |  |
|  | Residence ^c^ | 1.21 | 0.37 | 3.24** |  |  |  | 2.05 | 0.38 | 5.36** |  |  |
|  | Father’s age (year) | 0.04 | 0.05 | 0.74 |  |  |  | 0.15 | 0.05 | 2.87** |  |  |
|  | Mother’s age (year) | 0.19 | 0.05 | 3.67** |  |  |  | 0.04 | 0.05 | 0.77 |  |  |
|  | Father’s highest educational level ^d^ | -0.32 | 0.19 | -1.65 |  |  |  | -0.34 | 0.20 | -1.74 |  |  |
|  | Mother’s highest educational level ^d^ | -0.55 | 0.19 | -2.82** |  |  |  | -0.55 | 0.20 | -2.75** |  |  |
|  | Number of siblings ^e^ | -0.37 | 0.38 | -0.97 |  |  |  | 0.16 | 0.40 | 0.41 |  |  |
|  | Family monthly income ^f^ | 0.79 | 0.36 | 2.18* |  |  |  | 0.94 | 0.37 | 2.54* |  |  |
| 2 | PYD attributes | | | | | | | | | | | |
|  | Cognitive-behavioral competence | -5.37 | 0.23 | -23.84** | 0.14 | 161.1 |  | -6.06 | 0.21 | -28.88** | 0.19 | 228.8 |
|  | Prosocial attributes | -4.21 | 0.21 | -20.05** | 0.12 | 135.5 |  | -5.14 | 0.20 | -25.78** | 0.18 | 201.9 |
|  | Positive identity | -4.25 | 0.18 | -23.19** | 0.14 | 156.4 |  | -5.46 | 0.18 | -31.08** | 0.21 | 249.5 |
|  | General PYD | -6.86 | 0.24 | -29.13** | 0.18 | 204.2 |  | -7.29 | 0.22 | -33.49** | 0.22 | 237.2 |
|  | Total PYD | -6.88 | 0.24 | -28.56** | 0.17 | 199.2 |  | -7.60 | 0.22 | -34.16** | 0.23 | 281.2 |

Note. PYD = Positive youth development. In model 1, control variables were characteristics in Wave 1. In model 2, age, sex, residence, Mother’s age, Mother’s highest educational level and Family monthly income were statistically controlled in Young's Internet addiction test Wave 1 model; age, sex, residence, Father’s age, Mother’s highest educational level and Family monthly income were statistically controlled in Young's Internet addiction test Wave 2 model; measures of PYD in Wave 1 and Wave 2 were included as predictors to predict Internet addiction in Wave 1 and Wave 2, respectively. * p < .05; **p < .01.

^a^ 1 < 10 years old, 2 ≥ 10 years old; ^b^ 1 = male, 2 = female; ^c^ 1 = Urban, 2 = Rural; ^d^ 1=Primary school or lower, 2=Junior secondary school, 3=Senior Secondary school, 4=Diploma, 5=Undergraduate or higher; ^e^ 1 = Only-child, 2 = Non-only-child; ^f^ 1 = Gross Household Income (Monthly) was less than 8 K yuan, 2 = Gross Household Income (Monthly) was more than or equal to 8 K yuan.

Table S5. Longitudinal multiple regression analyses for Internet addiction (age as a binary variable)

| Model | Predictors | Young's Internet addiction test Wave 2 | | | | |  | Young's Internet addiction test change between Wave 1 and Wave 2 (Mean2- Mean1) | | | | |
| --- | --- | --- | --- | --- | --- | --- | --- | --- | --- | --- | --- | --- |
|  |  | β | SE | t | R^2^ | F |  | β | SE | t | R^2^ Change | F Change |
| 1 | Age (year) ^a^ | 4.19 | 0.34 | 12.33** | 0.37 | 388.8 |  | 1.91 | 0.38 | 5.08** | 0.005 | 4.97 |
|  | Sex ^b^ | -1.34 | 0.30 | -4.48** |  |  |  | 0.22 | 0.33 | 0.65 |  |  |
|  | Residence ^c^ | 1.37 | 0.32 | 4.28** |  |  |  | 0.84 | 0.36 | 2.35* |  |  |
|  | Father’s age (year) | 0.13 | 0.04 | 2.96** |  |  |  | 0.11 | 0.05 | 2.30* |  |  |
|  | Mother’s age (year) | -0.07 | 0.05 | -1.50 |  |  |  | -0.15 | 0.05 | -3.00** |  |  |
|  | Father’s highest educational level ^d^ | -0.16 | 0.17 | -0.99 |  |  |  | -0.02 | 0.19 | -0.13 |  |  |
|  | Mother’s highest educational level ^d^ | -0.24 | 0.17 | -1.42 |  |  |  | -0.002 | 0.19 | 0.009 |  |  |
|  | Number of siblings ^e^ | 0.38 | 0.33 | 1.13 |  |  |  | 0.54 | 0.37 | 1.45 |  |  |
|  | Family monthly income ^f^ | 0.50 | 0.31 | 1.60 |  |  |  | 0.15 | 0.34 | 0.44 |  |  |
|  | Young's Internet addiction test Wave 1 | 0.57 | 0.01 | 53.87** |  |  |  |  |  |  |  |  |
| 2 | **PYD attributes** |  |  |  |  |  |  |  |  |  |  |  |
|  | Cognitive-behavioral competence | -2.54 | 0.21 | -12.32** | 0.38 | 685.2 |  | 0.01 | 0.22 | 0.04 | 0.01 | 8.38 |
|  | Prosocial attributes | -2.30 | 0.19 | -12.24** | 0.38 | 684.7 |  | -0.31 | 0.21 | -1.50 | 0.01 | 8.83 |
|  | Positive identity | -2.42 | 0.17 | -14.59** | 0.39 | 701.4 |  | -0.38 | 0.18 | -2.11* | 0.01 | 9.27 |
|  | General PYD | -3.46 | 0.22 | -15.59** | 0.39 | 709.3 |  | -0.08 | 0.24 | -0.33 | 0.01 | 8.40 |
|  | Total PYD | -3.58 | 0.23 | -15.86** | 0.39 | 711.6 |  | -0.19 | 0.24 | -0.78 | 0.01 | 8.50 |

Note. PYD = Positive youth development. In model 1, control variables were characteristics in Wave 1. In model 2, age, sex, residence, father’s age and Young's Internet addiction test in Wave 1 were statistically controlled in Young's Internet addiction test Wave 2 model; age, residence, father’s age and mother’s age were statistically controlled in Young's Internet addiction test change model; measures of PYD in Wave 1 were included as predictors in the model separately. * p < .05; **p < .01.

^a^ 1 < 10 years old, 2 ≥ 10 years old; ^b^ 1 = male, 2 = female; ^c^ 1 = Urban, 2 = Rural; ^d^ 1=Primary school or lower, 2=Junior secondary school, 3=Senior Secondary school, 4=Diploma, 5=Undergraduate or higher; ^e^ 1 = Only-child, 2 = Non-only-child; ^f^ 1 = Gross Household Income (Monthly) was less than 8 K yuan, 2 = Gross Household Income (Monthly) was more than or equal to 8 K yuan.
